# Exploring risk factors for COVID-19 mortality and infection in care homes in the west of England: A mixed-methods study

**DOI:** 10.1177/13558196251344174

**Published:** 2025-06-03

**Authors:** Rebecca Wilson, Selin Siviş, Paul Scott, Jeremy Dixon, Karen Green, Judith Westcott, Alice Marriott, Jonathan Banks, Maria Theresa Redaniel

**Affiliations:** 1 The National Institute for Health and Care Research Applied Research Collaboration West (NIHR ARC West) & Population Health Sciences, University of Bristol, Bristol, UK; 2 1558 Bath and North East Somerset Council & College of Health, Science and Society, University of West England, Bath, UK; 3 School of Social Sciences, 2112 Cardiff University, Cardiff, UK; 4 1558 Bath and North East Somerset Council, Bath, UK; 5 NHS Dorset, Dorset, UK; 6 Wiltshire Council, Wiltshire, UK; 7 National Cancer Registry Ireland, Cork, Ireland

**Keywords:** care homes, COVID-19, England

## Abstract

**Objectives:**

Identify and explore risk factors associated with COVID-19 infection and mortality rates in care homes in the West of England and gain an understanding of challenges faced during the pandemic, how they were addressed and how care homes can be better equipped for future pandemics.

**Methods:**

A mixed-methods study combined observational analysis of numbers of infections and deaths with potential risk factors supported by semi-structured interviews. Thirty-three care homes within a single local authority (LA) in the West of England were included in the quantitative analysis and, in the qualitative study, five care homes were included, including those located outside the participating LA. The quantitative analysis assessed two outcomes: number of weekly COVID-19 cases and deaths between 31/08/2020 and 21/02/2021. Associations with potential care risk factors were analysed using Poisson regression. 14 interviews were conducted with care home staff in various roles between November 2022 and September 2023. Data were analysed thematically.

**Results:**

Care home size was associated with higher COVID-19 infection (large compared with small care homes: incidence rate ratio (IRR) = 12.60, 95% confidence interval (CI) 2.54 to 62.51) and mortality rates (large compared with small care homes: IRR = 16.48, 95% CI 0.81 to 335.88). Qualitative data revealed that care home managers recognized these risks and were focussed on the challenges of implementing infection control within the limitations of their buildings. The primary challenge identified was staff shortages, requiring care home staff to assume expanded responsibilities. There was no evidence of association between hospital discharges and COVID-19 cases (IRR = 0.45, 95% CI 0.11 to 1.83) or deaths (IRR = 0.61, 0.11 to 3.22). The qualitative data highlighted care home staff had feelings of separation and felt under-valued in relation to the wider health care sector. There was also concern that COVID-19 prevention measures prioritised infection control over the psycho-social welfare of residents.

**Conclusion:**

Research on the risk factors for infection spread and associated mortality should be prioritised to better protect care homes in future pandemics. This requires making routine data in social care more readily available for research purposes. Proactive planning for future pandemics, by care homes and local authorities, should recognise the individual nature of buildings and the needs of residents.

## Introduction

From early in the COVID-19 pandemic, it was evident that older adults, particularly those with comorbid health conditions, were vulnerable to COVID-19 and at greater risk of mortality.^1^ Care homes are high risk places for transmission,^2^ as they house large numbers of older people and have numerous visits from professionals, high numbers of mobile staff, and regular contact with hospitals.^3^ However, the UK policy-making agenda prioritised the National Health Service (NHS) over care homes which lacked equivalent levels of assistance or preparedness.^4^ They were initially considered low risk and designated as a place for discharged patients from hospital, as part of efforts to vacate hospital beds for newly admitted patients in critical conditions.^5,6^ However, care homes were disproportionately impacted by COVID-19, with cases estimated to be 13 times higher between March and June 2020 compared to the community.^7^

Policy measures were introduced to reduce risk of infection and deaths in care homes including the first lockdown in March 2020 which involved restricting visitor access and enforcing the use of personal protective equipment (PPE). Overall mortality risk rose during the second COVID wave (09/2020 - 04/2021) but remained below the peak levels of the first wave (03/2020 – 05/2020). This was due to enhanced infection prevention and control (IP&C) measures within care homes, higher levels of immunity among residents and demographic changes in the care home population. Additionally, a correlation was found between visitor restrictions, prompt health care access and mortality reduction. After the second wave, subsequent variants of COVID-19 saw infections leading to less severe disease among long-term care facility residents in England, likely due to high vaccine coverage, as well as natural immunity. However, managing breakthrough infections, particularly caused by variants, remained a persistent challenge for the sector.^8^ Recent studies have highlighted the role of environmental and structural factors in contributing to the COVID-19 infections in care homes including, purpose-built design, more bedrooms, and warmer temperatures identified as key contributors to increased transmission.^9^

Whilst these findings reflect a broader pattern observed across care homes in England, there were variations between COVID-19 mortality and infection rates across local authorities (LAs). LAs are a tier of UK local government which provide a key role in supporting care homes and ensuring they meet safety standards. The death rates in care homes in a particular LA in England were high compared with similar LAs during the pandemic’s second wave (September 2020 – April 2021).^10^ There were 523 COVID-19 deaths per 100,000 where the place of death was a care home for adults aged 75+, ranking 30 out of 312 lower tier LAs in England (where one had the highest rate).^7^ This was despite the region generally having lower rates of COVID-19 and the LA having COVID-19 and hospital death rates below the national average.^7^ In this LA, 50% of COVID-19 deaths were in care homes,^10^ compared with the English national average of 40% in the first wave and 26% in the second.^11^ We collaborated with this LA to gain an understanding of this disparity. The name of the LA is not disclosed to maintain the anonymity of care homes and confidentiality of participants.

We worked with the LA to examine potential risk factors contributing to resident COVID-19 infection and mortality rates and explored the challenges faced by the LA care homes during the pandemic, how they were addressed and how they can prepare for future pandemics. We consider structural, contextual, and processual factors, defined as: (a) *Structural* refers to the fundamental and long-lasting characteristics of care homes and their structure which affect their ability to manage infections; (b) *Contextual* refers to the situational, relational conditions and circumstances that influence infection rates within care homes; and (c) *Processual* refers to particular steps and actions taken to manage and reduce the risk of infection including the timeliness of response and adaption to IP&C policy requirements.

## Methods

This is a mixed methods study combining an observational study of potential risk factors associated with infection and death rates and a qualitative semi-structured interview study with care home staff.

### Quantitative study

#### Data sources

Anonymised data for the second COVID-19 wave from older adults’ care homes in the participating LA were provided by the COVID-19 Health Protection Manager. These included data from the commissioner for care homes, the contract review officer and IP&C team and were aggregated to care home level.

#### COVID-19 cases and deaths

Weekly data summarising COVID-19 cases and deaths from each care home between 31/08/2020-21/02/2021 were provided. COVID-19 cases were defined as a first positive COVID-19 test, not including reinfections and were based on Pillar 2 testing. COVID-19 deaths were defined as deaths where COVID-19 was mentioned anywhere on the death certificate.^12^

#### Covariates

Care home descriptive variables included care home speciality, type, ownership and size. An outbreak was defined as having two or more cases in a week. It was recorded whether, throughout the study period, care homes accepted admissions from acute or community hospitals to intermediate care beds (coded as: D2A - discharge to assess beds; Chi - beds in a care home acting as community hospital beds; or 3R - reablement: a temporary stay in a care home, community hospital, or standalone intermediate care facility) for short periods either before going home or into a long-term care home bed. Care home management was measured using four variables: high, medium or low engagement with commissioners and the IP&C team through, for example, voluntary forums, requests for information and offers of proactive support; the number of days per week the care home completed the capacity tracker; the length of time the manager had been in post (dichotomised as less than 1 year and 1 year or more); and whether or not the care home had high staff turnover (which captured whether there were frequent periods of lower than normal staffing levels due to recruitment and retention problems). GP involvement at the care home was described as high if regular face-to-face visits continued, medium if regular contact was through telephone or virtual calls or low if contact was only made when needed. Variables were also provided to indicate whether care homes received lateral flow tests on time or not and if their staff were in shared accommodation (either with staff from the same or another care home).

#### Statistical analysis

The number of days the capacity tracker was completed (used by the LA to monitor care home capacity), GP involvement, lateral flow tests (LFTs) received had missing data (N<5), which was assumed to be missing at random. Multiple imputation using chained equations was used to impute missing data with 100 imputations using Rubin’s rule.^13^ Following imputation, data were declared as time set panel data (care home ID was used as the panel variable).

The number of weekly COVID-19 cases and weekly deaths were regressed onto all covariates in univariable and multivariable Poisson regression models. Models used robust standard errors to account for clustering and were adjusted for calendar week. Care home ownership could not be included in the model due to collinearity and non-convergence issues. All analyses were done using Stata v17 software.

### Qualitative study

#### Recruitment

Initially, members of the participating LA contacted care homes in their area and invited contact with the research team. Following a low response rate, the research team contacted the regional comprehensive research network (CRN), encompassing the ENRICH network comprising care homes interested in research.^14^ The CRN contacted care homes in the West Country including areas outside the participating LA. This recruitment decision impacted the study’s original scope focusing on the participating LA area but enabled collection of qualitative data showing the regional care home experience during COVID-19. All targeted care homes received study information before participation. Staff were invited by care home managers and participation was voluntary.

#### Data collection

Interviews were audio-recorded and verbal consent was recorded. A topic guide, informed by the quantitative study along with existing research and reports, was developed to support the interviews. Data collection took place between November 2022 and September 2023.

#### Analysis

Transcripts were analysed thematically^15^ using interpretative, deductive and inductive analysis techniques. Analysis was an iterative process of close reading of the data, coding and elaboration of themes. SS and JB independently reviewed three interview transcripts to develop and agree a coding strategy in line with our research focus which combined investigation of risk factors examined in the quantitative analyses along with inductive codes. SS undertook full coding of the data, using NVivo 12 software. Codes, categories and thematic development were reviewed regularly by the project team, including LA collaborators who brought enriching perspectives and insights to the process.

## Findings

### Recruitment profile – quantitative study

Thirty-three care homes were included in the analysis. There were 290 COVID-19 cases across all care homes during the study period (weekly mean = 0.35, SD = 1.47) and 101 COVID-19 deaths (weekly mean = 0.12, SD = 0.57). The mean age of cases was 85.2 years (SD = 15.3) and 88.7 years for deaths (SD = 5.5); 71.4% of cases and 64.4% of deaths were female (see Online Supplement, Tables S1, S2 and S3).

### Recruitment profile – qualitative study

Five care homes were recruited across the region, two were from the participating LA. Fourteen semi-structured interviews were conducted with care home staff (see Table 1). Four interviews were conducted with care home managers (three of which were joint interviews where managers were accompanied by a care home administrator/business manager/finance administrator).

**Table 1.** Qualitative study – care home details.

Size of care home	Ownership type	Care home support provided	Interviewees and roles	ID
Small	Chain	Nursing, residential, and dementia care	Care home manager	1
			Senior administrator	2
Medium	Independent	Residential and nursing care	Care home manager	3
			Assistant manager	4
			Assistant manager	5
			Activity co-ordinator	6
Large	Independent	Nursing and dementia care	Care home manager	7
			Deputy manager	8
			Care coordinator	9
			Care coordinator	10
Large	Chain	Nursing and dementia care	Care home manager	11
			Senior administrator	12
			Care coordinator	13
Medium	Chain	Residential and dementia care	Care home manager	14

30 beds and under (Small); 60 beds and under (Medium); 61 and over (Large).

### Risk factor analyses

Associations between explanatory variables and COVID-19 cases and deaths in unadjusted and adjusted models are presented in Table 2.

**Table 2.** Univariate & multivariable Poisson regression models for Covid cases and deaths, adjusted for week and care home size, with robust standard errors, *N* = 33 care homes).

	Cases		Deaths	
Exposure variables	Univariate	Multivariable	Univariate	Multivariable
	Incidence ratio (95% CI)			
Type of care home				
Residential	1.00	1.00	1.00	1.00
Nursing	0.85 (0.34 to 2.12)	0.90 (0.34 to 2.36)	1.69 (0.69 to 4.12)	2.92 (0.53 to 16.16)
Care home specialty				
General	1.00	1.00	1.00	1.00
Dementia	1.05 (0.36 to 3.08)	0.82 (0.37 to 1.82)	1.42 (0.53 to 3.83)	1.06 (0.41 to 2.76)
Mixed	1.33 (0.62 to 2.87)	1.34 (0.41 to 4.38)	2.11 (0.89 to 5.02)	1.68 (0.55 to 5.09)
Care home ownership				
Chain/Council	1.00	1.00	1.00	1.00
Independent/Voluntary	0.78 (0.31 to 1.92)	0.76 (0.32 to 1.84)	0.67 (0.29 to 1.52)	0.71 (0.14 to 3.56)
Care home size				
Small	1.00	1.00	1.00	1.00
Medium	4.31 (1.62 to 11.49)*	3.93 (1.08 to 14.28)*	3.07 (1.03 to 9.13)*	1.12 (0.22 to 5.78)
Large	7.76 (2.86 to 21.11)*	12.60 (2.54 to 62.51)*	8.19 (2.73 to 24.62)*	16.48 (0.81 to 335.88)
Admission to D2A^a^ bed				
No	1.00	1.00	1.00	1.00
Yes	0.60 (0.32 to 1.12)	0.45 (0.11 to 1.83)	0.79 (0.43 to 1.44)	0.61 (0.11 to 3.22)
Manager in post				
One year or more	1.00	1.00	1.00	1.00
Less than a year	0.44 (0.24 to 0.80)*	0.19 (0.06 to 0.60)*	0.49 (0.25 to 0.93)*	0.08 (0.01 to 0.64)*
Engagement with local authority				
High	1.00	1.00	1.00	1.00
Medium	1.05 (0.43 to 2.59)	0.91 (0.23 to 3.62)	1.71 (0.73 to 4.01)	3.22 (0.73 to 14.33)
Low	0.52 (0.29 to 0.94)*	0.43 (0.12 to 1.55)	0.35 (0.15 to 0.80)*	0.22 (0.04 to 1.13)
Days per week capacity tracker completed				
5-6	1.00	1.00	1.00	1.00
3-4	1.32 (0.67 to 2.60)	1.45 (0.30 to 6.96)	1.02 (0.45 to 2.34)	4.07 (0.54 to 30.44)
1-2	1.58 (0.80 to 3.13)	2.78 (0.91 to 8.54)	1.35 (0.59 to 3.06)	10.57 (1.63 to 68.67)*
Staff turnover				
Stable staffing	1.00	1.00	1.00	1.00
Staffing issues	0.97 (0.54 to 1.73)	1.87 (0.80 to 4.33)	0.92 (0.48 to 1.74)	1.70 (0.57 to 4.91)
GP involvemen				
High	1.00	1.00	1.00	1.00
Medium	0.86 (0.43 to 1.72)	1.01 (0.27 to 3.75)	1.18 (0.49 to 2.81)	1.72 (0.27 to 11.02)
Low	0.98 (0.36 to 2.66)	1.12 (0.37 to 3.35)	1.32 (0.51 to 3.43)	1.54 (0.45 to 5.35)
Lateral flow tests (LFTs) received and used				
On time	1.00	1.00	1.00	1.00
Late	0.68 (0.34 to 1.37)	0.64 (0.23 to 1.79)	0.78 (0.34 to 1.81)	1.08 (0.21 to 5.61)
Staff in shared accommodation				
No	1.00	1.00	1.00	1.00
Yes from same home	1.20 (0.61 to 2.35)	0.93 (0.26 to 3.34)	0.97 (0.46 to 2.06)	0.79 (0.16 to 3.93)
Yes from another home	1.18 (0.58 to 2.41)	1.92 (0.59 to 6.21)	1.06 (0.50 to 2.24)	1.66 (0.26 to 10.77)

^a^D2A bed = discharge to assessed bed; *p < .05.

We explore and present our findings under two broad themes: (a) structural and contextual risk factors; and (b) processual risk factors. We combined structural and contextual risk factors because the situational and relational conditions of care homes are deeply connected to their long-lasting structural characteristics.

## Structural and contextual risk factors

### Physical infrastructure

In the quantitative analysis, care home size was associated with more cases and with more deaths (see Table 1). There was no evidence of association between other structural risk factors - including the type of care home (residential or nursing), care home speciality (general, dementia or mixed), and care home ownership (private, non-profit organisation, or government entity) - and the incidence of cases or deaths. From the qualitative study, some interviewees perceived that smaller care homes gave a greater ability to limit the virus. There was also a belief that the structure and age of the building were significant in controlling infections along with the capacity and spare bed capacity of the home:

“I think with it being a small home as well and because we were on three floors and they’re three completely separate floors they can be shut off, you can access each one from the outside, you don’t have to go through the house, I think that made it easier as well. Of course, being an old building didn’t help because trying to keep that sterile and everything it’s not like a hospital, you’ve got nooks and crannies everywhere that you’re trying to you know, make sure that they’re sterile” (1)

### Resident population

Qualitative findings demonstrated that the resident population played a role in shaping the care homes’ ability to isolate residents. Whilst the resident population profile is more related to the situational circumstances of care homes, it is crucial to recognise that care homes’ size, design, and layout, such as a lack of spacious communal areas, limited outdoor spaces, room configurations, or insufficient ventilation, can affect residents’ well-being, social interactions, and care experience. For instance, people with dementia did not necessarily comprehend the situation or IP&C measures such as isolation. This challenged the practical ability of care homes to implement these measures:

“Try to isolate someone that lives with dementia, and [they] can get upset and get anxious if he's alone in the room, and things like that, those were difficult times. (8)”

The focus of the pandemic was, understandably, IP&C measures. However, for the care homes the social and psychological impact on residents presented an equal challenge as IP&C:

“It was difficult because obviously we had to separate the residents, they had to stay in their rooms. They were isolated in a way because obviously we didn't have the staff who could sit with them for most of the day.” (4)

For some there was a tension between infection control, isolation and socialisation, which may have erred too far in preventing the spread of the virus at the expense of mental and social wellbeing:

“I learnt that isolating people in their rooms doesn’t work. It does not work because the virus goes round anyway […] that should be avoided for the residents' mental health and wellbeing.” (6)

### Workforce

In this study, workforce was investigated as a structural (e.g., longstanding staffing levels and workforce stability, employment conditions) and contextual risk factor (unexpected staffing gaps and emotional and psychological pressure on staff). The quantitative study investigated staff turnover in affecting infection and mortality and no association was observed (see Table 2). The qualitative data highlighted workforce as the most consistently reported risk factor by care home staff but in relation to staff shortages and welfare:

“Because when you’re a small care home and you may have five on shift and you have to have one or two people looking after particular COVID residents to try and prevent the infection spreading, that was challenging because we were having to maybe try and get more staff on duty.” (5)

Whilst the care homes experienced staff shortages prior to and during the pandemic, the nature of the work within care homes changed; the staff available were stretched impacting on their ability to manage infection control:

“The residents were having episodes of vomiting, they were having diarrhoea. We had to triple the collection of waste in a very short period of time. Everyone was working really hard, enter into rooms with PPE on, washing hands, everything. Yeah, the work increased so much, and it was difficult. I remember we had some team members that resigned at the time, because they were worried about having COVID, and they were worried that they would take it home.” (8)

Social and psychological challenges experienced by residents and staff during the lockdown period were exacerbated because of the increased levels of support and care needed:

“There was a lot more sort of stress, more, we had more issues with relatives questioning why they couldn’t come in and all that sort of thing, so it was the extra pressure […] when we had our outbreak so it was extremely stressful and extremely hard for staff, residents, I would say it was quite a traumatic time and I think a lot of the staff talk about, you know, post-traumatic stress.” (3)

The quantitative analysis identified an inverse association between the shorter time a care home manager had been in post and increased numbers of infections and deaths. However, corresponding qualitative data revealed a higher level of confidence and a perceived stronger skillset among managers in handling pandemic-associated challenges:

“Working in different sectors, working in different homes in the past, dealing with infections, dealing with chest infections, flu, all of those things that we’ve had in the past, it definitely, definitely helped me to cope a bit better with COVID. Again, having such a good team definitely, definitely helped me to cope with COVID”. (8)

## Processual risk factors

Processual risk factors concern the process involved in adapting to pandemic-induced challenges and describes interaction between care homes, COVID-19 policies, health care infrastructure, and administrative and governance infrastructure.

### Infection prevention and control (IP&C)

IP&C practices were already a critical aspect of care homes’ agendas prior to the pandemic and contributed to pandemic management and identified as a processual risk factor. Whilst there was no direct measure for the implementation of IP&C measures in the quantitative data, they showed that care homes undertook a range of IP&C practices in compliance with guidelines and tailored their capabilities and resources to maximise their effect. The care home manager below provides a good overview of the practices that were undertaken:

“We had the uniforms even being washed here on the premises, or if somebody wanted to take them home, they needed to take them in a plastic bag. So, in the changing room they would change their clothes, not wearing the uniform outside in the community and then coming at work. We purchased a mobile sink at the staff entrance so everybody would wash their hands as soon as they entered the door […] We increased the yellow bins probably to five times than the normal usage because the amount of PPE used was high to the roof [...]we needed to look into the breaks as well to make sure that we don’t exceed more than 14 members in the staff room at the same time than with the yellow tape […] We separated on tables to make sure that everybody’s at appropriate distance from one another, windows open at all times. We put air filters for communal areas because caring for people with dementia was not easy to confine them in one space.” (7)

Additionally, many care homes recognised the importance of introducing and enhancing IP&C training. These initiatives aimed to increase health literacy and awareness of preventative measures among care home staff and residents.

“I remember, the first time when we had to do the LFTs (lateral flow test), when they were first implemented, we had one day, we had the whole home coming to for the testing, it was myself and [anonymised] that did the training, we had test absolutely everyone. They were waiting in a line to be tested, and so on. As it progressed, we then trained them how to do it, they could do it at home and everything.” (8)

Whilst the use of PPE and other IP&C measures became normalised, as time went on, the commitment to their use started to wane:

“I think as we went along, people were picking what suited them in terms of infection control, and it's not always the matter of everyone doing exactly the same thing, going from one extreme to another.” (10)

For some, the standards of IP&C were perceived as impossible to implement and where some homes were able to organise their buildings to accommodate strict separation of COVID-19 cases this was not possible for others:

“This idea that you could try cohorts and keep COVID positive residents separate was a complete farce, it just didn’t work, and it was impossible. And almost cruel because you know, they don’t understand why they have to stay in their room, you know?” (3)

### Care homes and the health care infrastructure

Another processual risk factor was the care homes’ relationship with, and access to, wider health care services, including general practitioners (GPs), district nurses and secondary care. Although assigning a designated GP to every care home in the UK was mandatory, the relationship between care homes and GPs was strained during the pandemic. Quantitative data show the relationship of care homes with GPs was not associated with either case numbers or deaths. Whilst not directly attributing their relationship with wider health services as a contributing factor to care home deaths, care home participants expressed a sense of isolation which contributed to a perceived reduction in the quality of care they were able to deliver:

“One of the biggest things that we lost very quickly was our GP so they decided that they wouldn’t come into the home at all, we had no GP for eighteen months. So, because we’ve got our own nurses who are skilled, they just upskilled and sort of took over a lot of those roles that the GP would have done.” (13)

“We did experience the fact that it was slightly, or much more difficult to get in touch with the hospital, or with the ambulance crew members, it was harder for them to come to be seen.” (8)

Admissions from hospitals emerged as another processual risk factor as care homes were designated as a place to discharge patients from hospital. The quantitative investigation did not find associations between hospital admissions and infection or mortality rates. The qualitative data showed that care homes felt under pressure to admit patients from secondary care, leading them to assert their autonomy and resist taking hospital patients into their care home. This impacted on the relationship with the wider health care system and increased the feelings of alienation for care homes:

“There was a meeting with local authority, hospital and the care homes in the area. The consultant advised that [the] hospital expected to run out of capacity and therefore it would be seeking to discharge COVID-19 positive patients to the care homes for them to have palliative care in the care homes. But our decision was that we weren’t going to take any COVID-19 positive people. We would only accept if they had a COVID negative result.” (2)

### Relationship with local authorities

Collaboration with local authorities is a processual risk factor as receiving information on guidelines, support around IP&C training and resource access informs care home resilience during the pandemic. The quantitative investigation showed weak evidence of an association between lower engagement with the council and fewer cases and deaths, although these associations were attenuated in adjusted models. In the qualitative study, participants described a close working relationship with their LAs who provided information on guidelines, support around infection control training. They supported efforts to source PPE and facilitate grants made available by central government. The relationship with LAs was defined, largely, in positive and supportive terms which supported care homes in navigating the challenges of the pandemic:

“We had regular meetings with [LA] and other care homes and so they would be, you know, a way to you know introduce us to any new changes that the Government had put in place or Public Health England had put in place, and they would you know, help us to work out ways we can implement those policies and those changes. And also, obviously they started to supply the PPE for us so there was the NHS portal that we could get supplies and equipment.” (3)

Care homes would have liked more support from LAs in relation to staffing, identified as their biggest challenge during the pandemic. There were misconceptions about the potential of furloughed council staff (staff released from work duties on 80% salary) to act as an alternative staffing resource. This was not the case in the participating LAs but the perception that staff were not being used and could have been directed to support them reflected a communication breakdown:

“We would get these things from the local authority where we were told about all the measures and the testing but then when we were short staffed and we asked for help, all we were given was some advice on infection control.” (5)

### Capacity tracker

During the pandemic, care homes in England were required to use a capacity tracker (mandated 31 July 2022) which gave up-to-date information to LAs about their capacity to take patients from hospital along with information about COVID-19 infections and deaths. This tool marked a shift to new reporting structures within the sector, feeding into another processual aspect of pandemic management. Whilst in the quantitative investigation a potential trend was observed around use of the capacity tracker, the qualitative study revealed that the capacity tracker tended to be seen as a bureaucratic exercise with limited benefit, which undermined their relationship with the LA:

“I think it’s useful for them because they are collecting the information but it didn’t help us I don’t think in any way, I mean it was just an extra bit of work that we had to do that we did think, you know, why are we having to do this every day, putting this bit of information on and actually what help is it to anyone.” (3)

This view was not universal, and some care homes found completing the tracker gave them a perspective and overview of their own situation:

“Yeah, I'm still using it to this day. I think it was really, really helpful, putting all that information in there. It was really, really helpful for the local authority, for us as well, we can keep a good track of what's going on and we can see the information in there. So, we are still using it now.” (8)

## Discussion and conclusion

### Summary and comparison with literature

Care home size was associated with more cases and deaths, in line with previous research.^16^ In the quantitative analysis, care home size was directly correlated with weekly occupancy (number of beds occupied by residents), thus larger care homes had more residents which resulted in more cases and deaths. Further, larger care homes have more staff, which is a main route for introducing infection into a care home.^17^ The qualitative data indicate that care home managers placed more emphasis on organisation and team culture over the care home size. Whilst there was a perception that the situation may have been more manageable in smaller homes, this was overridden by other factors including: care home capacity, spare beds that gave the home more control over isolation practices; and the structure and age of the building.

Qualitative data identified staff shortages as the biggest challenge experienced by care homes. The sector was facing challenges -particularly in funding and recruitment- prior to the pandemic^5^ which were exacerbated throughout the pandemic with staff sickness and isolation.^18^ Care home staff were faced with taking on responsibilities previously belonging to other clinical service providers.^6^ Participants highlighted positive aspects associated with this including development of skills, however, research has shown burnout among care home staff was high.^19^

Care home specific guidance was limited, often confusing and contradictory, particularly when issued by multiple agencies.^19^ Concern for care homes, particularly in the early phase of the pandemic, was secondary to that for hospitals,^20^ despite lessons learned from previous epidemics.^21^ This perceived inferiority to hospitals was felt by care home staff, who felt undervalued compared with hospital staff.^6^ This was confirmed in our study which showed that care home staff felt alone and isolated as a provider of social and clinical care. It is notable that at least one care home felt empowered to resist taking COVID-19 patients from hospital while they were infected. There was also some evidence that the experience strained the relationship between care homes and secondary care. Staff described residents as feeling safer in care homes and reluctant to go to hospital and this has continued into the post-pandemic period.

We did not find an association between discharges to care homes from hospital and number of cases or deaths. Despite concern at the start of the first wave about the large number of individuals discharged from hospital to care homes to free up NHS hospital beds, often without testing,^5^ UK studies reported limited association between hospital discharge and cases in care homes, including when taking into account care home size and other care home factors.^22^ However, the reliance on care homes to receive patients discharged from hospital was thought to reflect a negligent attitude towards care homes,^5^ also evident in our findings.

Time the manager had been in post was associated with the number of cases and deaths in the quantitative analysis. While it was hypothesised that care homes with newer managers would be more likely to have more cases and deaths, due to inexperience and less familiarity with the care home, its staff and its residents, we found the opposite: that care homes with newer managers had fewer cases and deaths. Possible reasons for this were unmeasured confounders including behavioural qualities associated with being new in post. Manager experience was discussed with participants in the qualitative study and the limited data we had did not support the link between shorter length of service and lower death and infection rates. Managers placed high value on their length of service as an enabling quality in terms of infection control and communication. Engagement with the capacity tracker was discussed, but whilst not universal, the tool was seen as a bureaucratic exercise that took up staff time but with no or little benefit for the user. Yet, some care homes engaged positively with the tool and used it to keep track of their situation as providing data to the LA.

Prior to COVID-19 it was shown that the participating LA had one of the highest national percentages of people dying in care homes,^23^ and a lower percentage occurring in hospital.^24^ Reasons for this include lower emergency hospital admissions from care homes, compared with other LAs, a pattern that continued through the second wave of the pandemic, and the provision of GP care services in care homes, which aimed to support residents in their care homes and avoid escalation of care and hospital admission in the event of serious illness. Data also show that the LA had a higher proportion of nursing beds per 100 people aged of 75, further pointing to a vulnerable population with underlying health conditions, which was likely to better prepare care homes for managing residents when seriously ill and avoid unnecessary admissions.^25,26^ Our quantitative data indicated that this population was older (the mean age of the COVID-19 cases was 85 and of COVID-19 deaths was 89) and a high proportion of the homes were dementia care homes, suggesting that the care home residents were among the oldest and potentially most frail and vulnerable members of the population.

It is noteworthy that our study spans a relatively long time frame during which care homes were closed to visitors and vaccines were introduced. Variables, such as staff turnover and engagement with LAs, may have fluctuated over time and potentially affected outcomes. Previous research using national data showed that care home size (as measured by number of residents) did not change between waves one and two.^27^ This is important given the extensive changes in IP&C policies throughout the study period. These changes included but were not limited to: updates to quarantine and isolation policies, adjustments to hospital discharges and the establishment of designated settings, family visits, testing guidance (e.g., requirements for visiting professionals and introduction of lateral flow testing), financial support measures (e.g., Adult Social Care Infection Control and Testing Fund), the extension of free PPE, the implementation of mandatory vaccination policies, and an updated guide to vaccination boosters. The policy shifts may have introduced fluctuations in key variables and shaped the study’s findings. Importantly, as the quantitative data captured the second wave of the pandemic and the qualitative data beyond this, COVID-related deaths in care homes did decline from waves 1 to 2,^28^ most likely a reflection of the introduction of protective measures.

We conclude by reflecting on tensions between IP&C measures and the general wellbeing of residents. For some, the measures went too far in terms of reducing the social aspects of residents’ lives. There may be a case for care homes to be given greater levels of autonomy in managing these in the future.

### Limitations

We were only able to use anonymised aggregated data for our quantitative analysis. It is likely there are many unmeasured confounders (e.g., use of agency staff, vaccination status of residents, visitors or staff, staff infection rates, staff working across different sites) not adjusted for in the analysis. Patient-level data on usual place of care were not available so we were not able to infer whether patients died in their usual place of care. However, given that the participating LA had shown a consistent trend over the previous 10 years for higher rates of deaths in the usual place of residence, compared with the national average,^26^ it is likely that this continued throughout the pandemic, given that COVID-19 deaths in hospitals were lower than the national average in the region. We acknowledge that more robust analysis could have been done using patient-level or linked datasets. The relatively small number of care homes also meant that our analysis was underpowered. The number of residents at each care home was not provided (to preserve care homes’ anonymity), hence we do not have a denominator for our analysis. However, we were provided with care home size, which indicates the minimum and maximum number of residents at each care home and serves as a proxy for total number of residents.

For the qualitative study we were unable to recruit sufficient care homes to address the original research question directly and the lack of the resident voice in the work means we are missing a crucial perspective. The care homes where we did recruit were sampled by convenience rather than purposively, that is we included all those who were willing to participate rather than selecting to ensure that we covered a range of care home characteristics. We were able to interview care home managers and senior staff members but were unable to recruit from the full range of care home staff.

### Implications for practice

Barriers still exist in accessing research-ready data in social care, which is currently lacking in comparison with NHS data,^3^ despite the UK Government’s efforts to transition from a paper-based care planning system to a digital one, enabling non-clinical staff in social care settings to access necessary information and feed data into digital records in real time. Yet, these barriers are not unique to the UK; integrating social care and health care data remains a challenge on the policy agenda of many countries such as Belgium, France, and Switzerland owing to fragmentation in data collection and governance. In order to overcome data discrepancies, several pilot studies in the UK have been conducted to investigate the use of a minimum data set (MDS) in care homes, employing digital record systems to enhance care provision by linking care home data with resident health information from routine datasets. Recently there have been initiatives to link routinely collected datasets, including social care data, to enhance population health through the NHS Federated Data Platform.^29^ In this regard, the UK’s Federated Data Platform aligns with international practices in countries like New Zealand and Finland where strong governmental support and standardised data collections frameworks have positively shaped care outcomes.^30^ The inclusion of social care data in data infrastructures will provide health professionals, service managers and policymakers a better view of patient care. This can inform improvements in service delivery and patient experience of the health and care system.

The lower national excess deaths in the second wave in care homes^11^ show that lessons can be learnt and positive action taken to improve care homes’ chances in resisting the impact of an infection outbreak. There is no one-size-fits-all blueprint for care home improvements since each care home has unique needs and limitations. Thus there may be a case for developing, in partnership with local authorities, care home specific plans which recognise the scope and limitations of what can be achieved within particular buildings, and which could be updated regularly.

Our data highlight a range of complex challenges that went beyond IP&C including the psychosocial aspects of residents. A key aspect to diminish the feelings of abandonment and isolation is to support and maintain lines of communication especially around policy and guidance where multiple sources of information led to confusion and uncertainty. The data we have provided enable insights into the situation for care homes during the pandemic and offer pointers to future management during pandemics and outbreaks of severe infectious diseases.

## Supplemental Material

Supplemental Material - Exploring risk factors for COVID-19 mortality and infection in care homes in the west of England: A mixed-methods study Supplemental Material for Exploring risk factors for COVID-19 mortality and infection in care homes in the west of England: A mixed-methods study by Rebecca Wilson, Selin Sivis, Paul Scott, Jeremy Dixon, Karen Green, Judith Westcott, Alice Marriot, Jonathan Banks and Maria Theresa Redanie in Journal of Health Services Research & Policy.
