# Supplementary material for: Exploring risk factors for COVID-19 mortality and infection in care homes in the west of England: A mixed-methods study
Source: J Health Serv Res Policy. 2025 Jun 3;30(4):247–57. doi: 10.1177/13558196251344174 (PMC12411676; doi:10.1177/13558196251344174)
Supplement: Supplemental Material - Exploring risk factors for COVID-19 mortality and infection in care homes in the west of England: A mixed-methods study [file sj-pdf-1-hsr-10.1177_13558196251344174.pdf]

Online Supplement

Table S1: Characteristics of COVID-19 cases and deaths (1st September 2020 to 19th February 2021, 25 weeks)

Number of care homes included		33
Number of COVID cases in Wave 2		290
Mean age of COVID cases (SD)		85.22 (11.07)
Gender of COVID cases, N (%)[1]	Female	207 (71.38%)
	Male	83 (28.62%)
Number of COVID deaths in Wave 2		101
Mean age of COVID deaths (SD)		88.67 (5.47)
Gender of COVID deaths, N (%)	Female	65 (64.36%)
	Male	36 (35.64%)

[1] Older adults care home populations have a higher ratio of females to males (Office for National Statistics, 2020)

Table S2: Weekly mean COVID cases and deaths for study period (1st September 2020 to 19th February 2021, 25 weeks)

	Mean (SD)
Mean COVID-19 infections per Care home per week	0.35 (1.47)<br>[range 0 – 16]
Mean COVID-19 deaths per Care home per week	0.12 (0.57)<br>[range 0 – 6]

Table S3: quantitative study - care home characteristics

Care home characteristics (N=33)		N (%)
Care home specialty	General	19 (57.6)
	Dementia	6 (18.2)
	Mixed	8 (24.2)
Type of Care home	Residential	15 (45.5)
	Nursing	18 (54.6)
Care home Ownership	Chain / Council	20 (60.6)
	Independent / Voluntary	13 (39.4)
Care home size	Small (≤29 beds)	8 (24.2)
	Medium (30-59 beds)	19 (57.6)
	Large (≥60 beds)	6 (18.2)
Wave 2 outbreak?	No	14 (42.4)
	Yes	17 (51.5)
	Unknown	2 (6.1)
Admissions from hospital into D2A, 3R or Chi beds	No	9 (27.3)
	Yes	24 (72.7)
Manager in post	One year or more	24 (72.7)
	Less than a year	9 (27.3)
Engagement with local authority	High	18 (54.6)
	Medium	8 (24.2)
	Low	7 (21.2)
Days per week capacity tracker completed (N=31)	5-6	7 (22.6)
	3-4	14 (45.2)
	1-2	10 (32.3)
Staff turnover	Stable staffing	15 (45.5)
	Staffing issues	18 (54.6)
Support from provider/area manager (N=30)	High	16 (53.3)
	Medium	6 (20.0)
	Low	8 (26.7)
GP involvement[1]	High	7 (21.2)
	Medium	20 (60.6)
	Low	<5 (<10.0)
	Unknown	<5 (<10.0)
LFTs received and used	On time	10 (30.3)
	Late	13 (39.4)
	Unknown	10 (30.3)
Staff in shared accommodation	No	16 (48.5)
	Yes from same home	10 (30.3)
	Yes from another home	7 (21.2)

[1] Small (<5) cell counts suppressed.
